# Author Correction: Community assessment of methods to deconvolve cellular composition from bulk gene expression

**DOI:** 10.1038/s41467-024-53843-9

**Published:** 2024-11-12

**Authors:** Brian S. White, Aurélien de Reyniès, Aaron M. Newman, Joshua J. Waterfall, Andrew Lamb, Florent Petitprez, Yating Lin, Rongshan Yu, Martin E. Guerrero-Gimenez, Sergii Domanskyi, Gianni Monaco, Verena Chung, Jineta Banerjee, Daniel Derrick, Alberto Valdeolivas, Haojun Li, Xu Xiao, Shun Wang, Frank Zheng, Wenxian Yang, Carlos A. Catania, Benjamin J. Lang, Thomas J. Bertus, Carlo Piermarocchi, Francesca P. Caruso, Michele Ceccarelli, Thomas Yu, Xindi Guo, Julie Bletz, John Coller, Holden Maecker, Caroline Duault, Vida Shokoohi, Shailja Patel, Joanna E. Liliental, Stockard Simon, Tumor Deconvolution DREAM Challenge consortium, Aaron M. Newman, Joshua J. Waterfall, Andrew Lamb, Florent Petitprez, Yating Lin, Rongshan Yu, Martin E. Guerrero-Gimenez, Sergii Domanskyi, Gianni Monaco, Verena Chung, Jineta Banerjee, Daniel Derrick, Alberto Valdeolivas, Haojun Li, Xu Xiao, Shun Wang, Frank Zheng, Wenxian Yang, Carlos A. Catania, Benjamin J. Lang, Thomas J. Bertus, Carlo Piermarocchi, Francesca P. Caruso, Michele Ceccarelli, Thomas Yu, Xindi Guo, Julie Bletz, John Coller, Holden Maecker, Caroline Duault, Vida Shokoohi, Shailja Patel, Joanna E. Liliental, Stockard Simon, Brian S. White, Aurélien de Reyniès, Aashi Jain, Shreya Mishra, Vibhor Kumar, Jiajie Peng, Lu Han, Gonzalo H. Otazu, Austin Meadows, Patrick J. Danaher, Maria K. Jaakkola, Laura L. Elo, Julien Racle, David Gfeller, Dani Livne, Sol Efroni, Tom Snir, Oliver M. Cast, Martin L. Miller, Dominique-Laurent Couturier, Wennan Chang, Sha Cao, Chi Zhang, Dominik J. Otto, Kristin Reiche, Christoph Kämpf, Michael Rade, Carolin Schimmelpfennig, Markus Kreuz, Alexander Scholz, Julio Saez-Rodriguez, Laura M. Heiser, Justin Guinney, Andrew J. Gentles, Julio Saez-Rodriguez, Laura M. Heiser, Justin Guinney, Andrew J. Gentles

**Affiliations:** 1 Sage Bionetworks, Seattle, WA USA; 2 Centre de Recherche des Cordeliers, INSERM U1138, Université Paris Cité, Paris, France; 3 Institute for Stem Cell Biology and Regenerative Medicine, Stanford University, Stanford, CA USA; 4 Department of Biomedical Data Science, Stanford University, Stanford, CA USA; 5 INSERM U830 and Translational Research Department, Institut Curie, PSL Research University, Paris, France; 6 Programme Cartes d’Identité des Tumeurs, Ligue Nationale Contre le Cancer, Paris, France; 7 MRC Centre for Reproductive Health, the Queen’s Medical Research Institute, University of Edinburgh, Edinburgh, United Kingdom; 8 Xiamen University, Xiamen, Fujian, China; 9 Institute of Biochemistry and Biotechnology, School of Medicine, National University of Cuyo, Mendoza, Argentina; 10 Michigan State University, East Lansing, MI USA; 11 BIOGEM Institute of Molecular Biology and Genetics, Ariano Irpino, AV Italy; 12 Department of Biomedical Engineering, Knight Cancer Institute, Oregon Health & Science University, Portland, OR USA; 13 Heidelberg University, Faculty of Medicine, Heidelberg, Germany; 14 Heidelberg University Hospital, Institute for Computational Biomedicine, Bioquant, Heidelberg, Germany; 15 Aginome Scientific, Xiamen, Fujian, China; 16 Department of Pathology, Cancer Hospital, Chinese Aacdemy of Medical Science, Beijing, China; 17 AmoyDx, Xiamen, Fujian, China; 18 Laboratory of Intelligent Systems (LABSIN), Engineering School, National University of Cuyo, Mendoza, Argentina; 19 Department of Radiation Oncology, Beth Israel Deaconess Medical Center, Harvard Medical School, Boston, MA USA; 20 Department of Electrical Engineering and Information Technology (DIETI), University of Naples “Federico II”, Ariano Irpino, AV Italy; 21 Sylvester Comprehensive Cancer Center, Department of Public Health Sciences, University of Miami Miller School of Medicine, Miami, Florida USA; 22 Stanford Functional Genomics Facility, Stanford University School of Medicine, Stanford, CA USA; 23 Institute for Immunity, Transplantation, and Infection, Stanford University School of Medicine, Stanford, CA USA; 24 Translational Applications Service Center, Stanford University School of Medicine, Stanford, CA USA; 25 Department of Medicine, Stanford University School of Medicine, Stanford, CA USA; 26 Department of Pathology, Stanford University, Stanford, CA USA; 27 Department of Computer Science, Indraprastha Institute of Information Technology, Okhla Ph-3, New Delhi, India; 28 School of Computer Science, Northwestern Polytechnical University, Xi’an, Shaanxi China; 29 Center for Biomedical Innovation, New York Institute of Technology, College of Osteopathic Medicine, New York, USA; 30 Icahn School of Medicine at Mount Sinai, New York, USA; 31 NanoString Technologies, Seattle, WA USA; 32 Turku Bioscience Centre, University of Turku and Åbo Akademi University, Turku, Finland; 33 Department of Mathematics and Statistics, University of Turku, Turku, Finland; 34 Institute of Biomedicine, University of Turku, Turku, Finland; 35 Department of Oncology UNIL CHUV, Ludwig Institute for Cancer Research, University of Lausanne, Lausanne, Switzerland; 36 Swiss Institute of Bioinformatics (SIB), Lausanne, Switzerland; 37 The Mina & Everard Goodman Faculty of Life Sciences, Bar-Ilan University, Ramat Gan, Israel; 38 Cancer Research UK, Cambridge Institute, University of Cambridge, Cambridge, UK; 39 Electrical and Computer Engineering, Purdue University, West Lafayette, IN USA; 40 Department of Biostatistics, Indiana University, School of Medicine, Indianapolis, IN USA; 41 Medical and Molecular Genetics, Indiana University School of Medicine, Indianapolis, IN USA; 42 Department of Diagnostics, Fraunhofer Institute for Cell Therapy and Immunology, Perlickstraße 1, 04103 Leipzig, Germany; 43 Institute for Clinical Immunology, Leipzig University, Johannisallee 30, 04103 Leipzig, Germany; 44 Present Address: The Jackson Laboratory for Genomic Medicine, Farmington, CT USA

**Keywords:** Computational models, Cancer genomics, Immunology, Bioinformatics, Cancer microenvironment

Correction to: *Nature Communications* 10.1038/s41467-024-50618-0, published online 27 August 2024

The original version of this Article omitted from the Tumor Deconvolution DREAM Challenge consortium the members Aashi Jain, Shreya Mishra, and Vibhor Kumar from Department of Computer Science, Indraprastha Institute of Information Technology, Okhla Ph-3, New Delhi, India, Jiajie Peng and Lu Han from School of Computer Science, Northwestern Polytechnical University, Xi’an, Shaanxi, China, Gonzalo H Otazu from Center for Biomedical Innovation, New York Institute of Technology, College of Osteopathic Medicine, New York, USA, Austin Meadows from Icahn School of Medicine at Mount Sinai, New York, USA, Patrick J Danaher from NanoString Technologies, Seattle, WA, USA, Maria K Jaakkola from Turku Bioscience Centre, University of Turku and Åbo Akademi University, Finland and Department of Mathematics and Statistics, University of Turku, Finland, Laura L Elo from Turku Bioscience Centre, University of Turku and Åbo Akademi University, Finland and Institute of Biomedicine, University of Turku, Finland, Julien Racle and David Gfeller from Department of Oncology UNIL CHUV, Ludwig Institute for Cancer Research, University of Lausanne, Lausanne, Switzerland and Swiss Institute of Bioinformatics (SIB), Lausanne, Switzerland, Dani Livne, Sol Efroni, and Tom Snir from The Mina & Everard Goodman Faculty of Life Sciences, Bar-Ilan University, Israel, Oliver M Cast, Martin L Miller, and Dominique-Laurent Couturier from Cancer Research UK, Cambridge Institute, University of Cambridge, UK, Wennan Chang from Electrical and Computer Engineering, Purdue University, West Lafayette, IN, USA, Sha Cao from Department of Biostatistics, Indiana University, School of Medicine, Indianapolis, IN, USA, Chi Zhang from Medical and Molecular Genetics, Indiana University School of Medicine, Indianapolis, IN, USA, Dominik J Otto and Kristin Reiche from Department of Diagnostics, Fraunhofer Institute for Cell Therapy and Immunology, Perlickstraße 1, 04103 Leipzig, Germany and Institute for Clinical Immunology, Leipzig University, Johannisallee 30, 04103 Leipzig, Germany, Christoph Kämpf, Michael Rade, Carolin Schimmelpfennig, Markus Kreuz, and Alexander Scholz from Department of Diagnostics, Fraunhofer Institute for Cell Therapy and Immunology, Perlickstraße 1, 04103 Leipzig, Germany.

This has been corrected in both the PDF and HTML versions of the Article.

