# Supplementary material for: Community assessment of methods to deconvolve cellular composition from bulk gene expression
Source: Nat Commun. 2024 Aug 27;15:7362. doi: 10.1038/s41467-024-50618-0 (PMC11350143; doi:10.1038/s41467-024-50618-0)
Supplement: Supplementary file 3 — Description of Additional Supplementary Files [file 41467_2024_50618_MOESM3_ESM.pdf]

Description of Additional Supplementary Files:

Supplementary Data 1: Isolated cell populations used in Challenge

Supplementary Data 2: Quality assessment of isolated cell popluations

Supplementary Data 3: Computationally defined biologically constrained admixtures (favoring Stem Express in selecting from biological replicates)

Supplementary Data 4: Computationally defined unconstrained admixtures (favoring Stem Express in selecting from biological replicates)

Supplementary Data 5: Computationally defined biologically constrained admixtures (favoring All Cells in selecting from biological replicates)

Supplementary Data 6: Computationally defined unconstrained admixtures (favoring All Cells in selecting from biological replicates)

Supplementary Data 7: Experimentally generated admixtures (favoring Stem Express in selecting from biological replicates). Biologically constrained admixtures designated as "BM." Unconstrained admixtures designated as "RM."

Supplementary Data 8: Experimentally generated admixtures (favoring All Cells in selecting from biological replicates). Biologically constrained admixtures designated as "BM." Unconstrained admixtures designated as "RM."

Supplementary Data 9: Curated GEO expression array samples provided to participants for method training

Supplementary Data 10: Curated GEO RNA-seq samples provided to participants for method training

Supplementary Data 11: Execution times for methods in Challenge validation phase

Supplementary Data 12: Annotated bulk samples used for training Aginome-XMU deconvolution method

Supplementary Data 13: Annotated bulk samples used for training DA_505 deconvolution method

Supplementary Data 14: Annotated bulk samples used for training mitten_TDC19 deconvolution method

Supplementary Data 15: Annotated bulk samples used for training Biogem deconvolution method

Supplementary Data 16: Dataset-specific Pearson correlation statistical differences. t test used to assess p-values, which are reported as raw one (one.sided.pval) or two-sided (two.sided.pval) p-values, as well as one (one.sided.pval.holm) and two-sided (two.sided.pval.holm) p-values following correction with Holm-Bonferroni.

Supplementary Data 17: Translation between Pelka et al. and Challenge cell types
