# Supplementary material for: Community assessment of methods to deconvolve cellular composition from bulk gene expression
Source: Nat Commun. 2024 Aug 27;15:7362. doi: 10.1038/s41467-024-50618-0 (PMC11350143; doi:10.1038/s41467-024-50618-0)
Supplement: Supplementary file 5 — Reporting Summary [file 41467_2024_50618_MOESM5_ESM.pdf]

nature portfolio

Corresponding author(s): Andrew Gentles

Last updated by author(s): May 3, 2024

Reporting Summary

Nature Portfolio wishes to improve the reproducibility of the work that we publish. This form provides structure for consistency and transparency in reporting. For further information on Nature Portfolio policies, see our Editorial Policies and the Editorial Policy Checklist.

Statistics

For all statistical analyses, confirm that the following items are present in the figure legend, table legend, main text, or Methods section.

n/a	Confirmed	
☐	☒	The exact sample size (*n*) for each experimental group/condition, given as a discrete number and unit of measurement
☐	☒	A statement on whether measurements were taken from distinct samples or whether the same sample was measured repeatedly
☐	☒	The statistical test(s) used AND whether they are one- or two-sided<br>*Only common tests should be described solely by name; describe more complex techniques in the Methods section.*
☒	☐	A description of all covariates tested
☒	☐	A description of any assumptions or corrections, such as tests of normality and adjustment for multiple comparisons
☐	☒	A full description of the statistical parameters including central tendency (e.g. means) or other basic estimates (e.g. regression coefficient) AND variation (e.g. standard deviation) or associated estimates of uncertainty (e.g. confidence intervals)
☐	☒	For null hypothesis testing, the test statistic (e.g. *F*, *t*, *r*) with confidence intervals, effect sizes, degrees of freedom and *P* value noted<br>*Give P values as exact values whenever suitable.*
☒	☐	For Bayesian analysis, information on the choice of priors and Markov chain Monte Carlo settings
☒	☐	For hierarchical and complex designs, identification of the appropriate level for tests and full reporting of outcomes
☐	☒	Estimates of effect sizes (e.g. Cohen's *d*, Pearson's *r*), indicating how they were calculated

Our web collection on statistics for biologists contains articles on many of the points above.

Software and code

Policy information about availability of computer code

Data collection	No software was used for data collection.
Data analysis	All analyses were performed using R statistical software. Pearson and Spearman correlations were calculated with car.test and two-sided Wilcoxon tests were performed using compare_means.

For manuscripts utilizing custom algorithms or software that are central to the research but not yet described in published literature, software must be made available to editors and reviewers. We strongly encourage code deposition in a community repository (e.g. GitHub). See the Nature Portfolio guidelines for submitting code & software for further information.

Data

Policy information about availability of data

All manuscripts must include a data availability statement. This statement should provide the following information, where applicable:

- Accession codes, unique identifiers, or web links for publicly available datasets
- A description of any restrictions on data availability
- For clinical datasets or third party data, please ensure that the statement adheres to our policy

The raw RNA-seq data generated in this study have been deposited in the NCBI's Sequence Read Archive (SRA) database under accession code SRP365686 [https://www.ncbi.nlm.nih.gov/sra/?term=SRP365686]. The processed RNA-seq data have been deposited in the NCBI's Gene Expression Omnibus89 (GEO) database under accession code GSE199324 [https://www.ncbi.nlm.nih.gov/geo/query/acc.cgi?acc=GSE199324]. Additionally, the raw and processed RNA-seq data are hosted on the Synapse data-sharing platform at syn21557721 and syn21571479, respectively. Source data are provided with this paper and are available 10.5281/

zenodo.11106980. Raw count data and metadata generated by the Wu et al. BRCA single-cell study34 can be downloaded from the Broad Single Cell Portal [https://singlecell.broadinstitute.org/single_cell/study/SCP1039/a-single-cell-and-spatially-resolved-atlas-of-human-breast-cancers]. Raw data generated by the Pelka et al. CRC single-cell study35 are available under GEO accession code GSE178341 [https://www.ncbi.nlm.nih.gov/geo/query/acc.cgi?acc=GSE178341].

Research involving human participants, their data, or biological material

Policy information about studies with human participants or human data. See also policy information about sex, gender (identity/presentation), and sexual orientation and race, ethnicity and racism.

Reporting on sex and gender	Information on sex and gender was not collected or analyzed in this study, which does not consider correlates related to individual patients.
Reporting on race, ethnicity, or other socially relevant groupings	Information on race and ethnicity was not considered in this study.
Population characteristics	Information on race and ethnicity was not considered in this study.
Recruitment	Individuals were not recruited to this study, previously biobanked specimens were utilized.
Ethics oversight	Fresh whole blood was obtained from the Stanford Blood Center, in accordance with procedures approved by the Stanford Institutional Review Board (IRB).

Note that full information on the approval of the study protocol must also be provided in the manuscript.

Field-specific reporting

Please select the one below that is the best fit for your research. If you are not sure, read the appropriate sections before making your selection.

[x] Life sciences [ ] Behavioural & social sciences [ ] Ecological, evolutionary & environmental sciences

For a reference copy of the document with all sections, see nature.com/documents/nr-reporting-summary-flat.pdf

Life sciences study design

All studies must disclose on these points even when the disclosure is negative.

Sample size	1000 bootstraps were used to compare methods. A sample size of 1000 was chosen so that intra-method variance across bootstraps was small (i.e., relative to inter-method variance).
Data exclusions	No data were excluded.
Replication	Cell populations were generally derived from two biological replicates (often from different vendors). Exceptions were memory B cells, which were not replicated owing to poor RNA integrity of the second sample. Further, fibroblasts, endothelial cells, and colon and breast cancer cells were not replicated, as they were derived from cell lines.
Randomization	The experiments were not randomized, since there were no experimental groups in our study and randomization was not relevant.
Blinding	The investigators were not blinded during the study, as there were no group allocations.

Reporting for specific materials, systems and methods

We require information from authors about some types of materials, experimental systems and methods used in many studies. Here, indicate whether each material, system or method listed is relevant to your study. If you are not sure if a list item applies to your research, read the appropriate section before selecting a response.

Materials & experimental systems

n/a	Involved in the study	
x		Antibodies
	x	Eukaryotic cell lines
x		Palaeontology and archaeology
x		Animals and other organisms
x		Clinical data
x		Dual use research of concern
x		Plants

Methods

n/a	Involved in the study	
x		ChIP-seq
x		Flow cytometry
x		MRI-based neuroimaging

Eukaryotic cell lines

Policy information about cell lines and Sex and Gender in Research

Cell line source(s)	ATCC (HTB-38, HTB-121, PCS-201-012, PCS-100-11)
Authentication	None
Mycoplasma contamination	Not tested.
Commonly misidentified lines (See ICLAC register)	No misidentified lines used.

Plants

Seed stocks	*Report on the source of all seed stocks or other plant material used. If applicable, state the seed stock centre and catalogue number. If plant specimens were collected from the field, describe the collection location, date and sampling procedures.*
Novel plant genotypes	*Describe the methods by which all novel plant genotypes were produced. This includes those generated by transgenic approaches, gene editing, chemical/radiation-based mutagenesis and hybridization. For transgenic lines, describe the transformation method, the number of independent lines analyzed and the generation upon which experiments were performed. For gene-edited lines, describe the editor used, the endogenous sequence targeted for editing, the targeting guide RNA sequence (if applicable) and how the editor was applied.*
Authentication	*Describe any authentication procedures for each seed stock used or novel genotype generated. Describe any experiments used to assess the effect of a mutation and, where applicable, how potential secondary effects (e.g. second site T-DNA insertions, mosiacism, off-target gene editing) were examined.*
